# Developmental dynamics of *Ambystoma tigrinum *in a changing landscape

**DOI:** 10.1186/1472-6785-10-10

**Published:** 2010-04-03

**Authors:** Sarah K McMenamin, Elizabeth A Hadly

**Affiliations:** 1 Department of Biology, Stanford University, Stanford CA 94305-5020, USA; 2 Present address: Department of Biology, University of Washington, Box 351800, Seattle WA 98195-1800, USA

## Abstract

**Background:**

Loss of pond habitat is catastrophic to aquatic larval amphibians, but even reduction in the amount of time a breeding site holds water (hydroperiod) can influence amphibian development and limit reproductive success. Using the landscape variation of a glacial valley in the Greater Yellowstone Ecosystem as the context for a natural experiment, we examined variation in growth pattern and life history of the salamander *Ambystoma tigrinum melanostictum *and determined how these developmental characteristics varied with hydroperiod over several summers.

**Results:**

In ponds that dried early in the season, maximum larval size was reduced relative to the sizes achieved in permanent ponds. Ephemeral ponds were associated with early metamorphosis at small body sizes, while permanent ponds facilitated longer larval periods and later metamorphosis. Paedomorphosis resulted from indefinite metamorphic postponement, and was identified only in the most permanent environments. Patterns of growth and allometry were similar between ponds with different hydroperiods, but considerable life history variation was derived from modulating the timing of and size at metamorphosis. Considering maximum rates of growth and inferring the minimum size at metamorphosis across 25 ponds over the course of three years, we calculated that hydroperiods longer than three months are necessary to support these populations through metamorphosis and/or reproductive maturity.

**Conclusions:**

Landscape heterogeneity fosters life history variation in this natural population. Modulation of the complex ambystomatid life cycle allows this species to survive in unpredictable environments, but current trends towards rapid pond drying will promote metamorphosis at smaller sizes and could eliminate the paedomorphic phenotype from this region. Metamorphosis at small size is has been linked to altered fitness traits, including reduced survival and fecundity. Thus, widespread environmental truncation of larval periods may lead to decreased population persistence. We found that the hydroperiods of many ponds in this region are now shorter than the developmental period required for larvae to reach the minimum size for metamorphosis; these locations serve as reproductive sinks that may be detrimental for persistence of the species in the region.

## Background

Phenotype is determined by the interaction of genotype and developmental environment, but the influence of environment on variation within species is sometimes under-emphasized. While canalization produces consistent phenotypic outcomes under a variety of conditions [1], phenotypic plasticity produces developmental outcomes that vary with environment [2]. Vertebrate development, and particularly development of larval amphibians, can be modified by many factors, including temperature [3,4], density [5-7] and resource availability [4,8,9]. These extrinsic signals are transduced into endocrinological signals that ultimately control rates of growth and timing of developmental events [8,10].

The optimal phenology of developmental events depends on ecological context and conditions [11], and plasticity in developmental timing can allow organisms to maximally exploit a range of unpredictable environments [12-15]. When a complex life cycle involves larval and adult stages that inhabit different ecological niches, species can particularly benefit from plasticity in the timing of the metamorphic transition. Most amphibians require an aquatic environment during larval development, and developmental plasticity allows populations to maximally exploit both permanent and ephemeral larval habitats, with rapidly drying aquatic habitats associated with rapid metamorphosis [16-19]. However, the timing of metamorphosis affects phenotype, survival, age at first reproduction and reproductive success, and shorter larval periods may lead to decreased lifetime fecundity [5,18,20]. Moreover, there is thought to be a minimum size at which metamorphosis can occur (SVL_m_) [11,12,21,22], and if larvae cannot grow fast enough to reach SVL_m _before an environment becomes uninhabitable, larvae perish before recruitment. There is thus a apparent trade-off between metamorphosis at large sizes (maximizing terrestrial survival and fecundity) and metamorphosis at small sizes (minimizing larval mortality) [22,23].

If the resources necessary to support growth to SVL_m _are not available, individuals of facultatively paedomorphic species are hypothesized to become reproductively mature in a larval form ("best of a bad lot" progenic paedomorphosis) [21,24]. At the opposite end of the plasticity spectrum, if the larval environment is particularly resource-rich, individuals may permanently remain in the larval niche, becoming paedomorphic even after SVL_m _is reached. These "paedomorph advantage" individuals are expected to show higher fecundity than both progenic paedomorphs and metamorphosed individuals [21,24].

With alternative developmental strategies expressed under different environmental conditions, habitat heterogeneity across geographic space promotes diversity in phenotype and life history [16,25,26]. Developmental responses to environmental conditions are observed to vary through time as well, and millennial-scale climatic events catalyzed changes in the growth and developmental timing of *Ambystoma tigrinum melanostictum *populations in Yellowstone National Park (YNP) [27].

In this study, we characterized life histories, metamorphic timing and patterns of growth in *A. t. melanostictum *populations across a glacially modified landscape in the Greater Yellowstone Ecosystem (GYE). Recent evidence that ponds in this landscape are changing to the detriment of local amphibian populations [28] makes investigating the relationships between population status and landscape conditions particularly urgent. Focusing on populations in 25 heterogeneous pond environments, we characterized (1) the rates of larval growth, (2) the maximum sizes attained by larvae and the minimum sizes at maturity, and (3) the patterns of allometric growth as each varied with the hydroperiods of the different ponds. Considering the fitness associations of different developmental strategies and the limits of developmental flexibility of this species, we discuss the long-term implications of the climatic and environmental changes taking place in northern YNP.

## Results

During the summer months of 2006-2008, we measured 609 *A. t. melanostictum *individuals captured from 25 pond environments in the GYE, including 22 within YNP (Additional file 1). Of the individuals sampled, 519 (85%) were aquatic larvae, 71 (12%) were terrestrial metamorphs and 19 (3%) were mature aquatic paedomorphs.

### Growth rate

**Figure 1:**
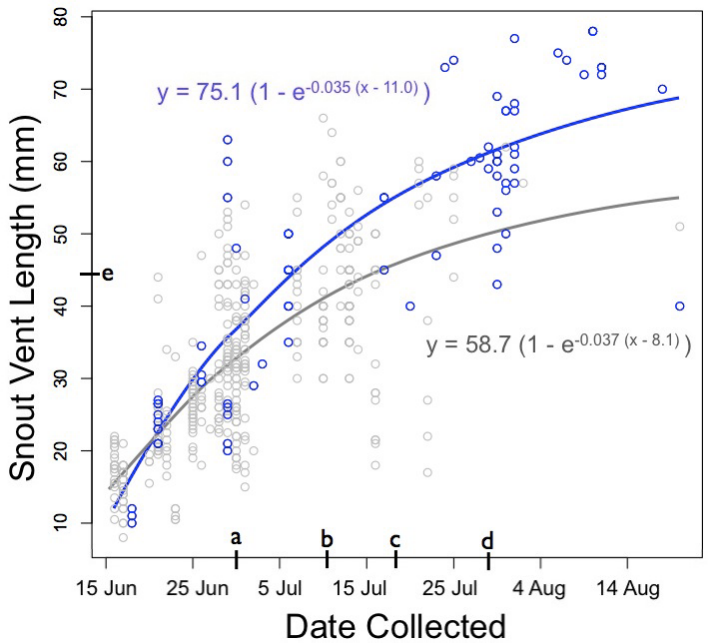
**Growth of salamanders over time during summers 2006-2008**. *A. t. melanostictum *larvae collected in YNP during the summers of 2006-2008. Blue rings denote larvae collected from permanent environments (n = 81); gray rings denote larvae collected from ephemeral environments (n = 405). Curves show Von Bertalanffy models of growth: growth of larvae from permanent environments shown with blue line; growth of larvae from ephemeral ponds shown with gray. On the y axis, **a **shows date when the first ephemeral pond dried (30 June); **b **shows first date that an emerging metamorph was found in an ephemeral pond (10 July); **c **shows earliest date that an emerging metamorph was found in a permanent pond (19 July); **d **shows earliest date a recently mature paedomorph was found in a permanent pond (30 July). On the y axis, **e **shows the size of the smallest metamorph captured (45 mm ≅ SVL_m_).

We modelled the rate of larval growth in ephemeral (n = 405 larvae) and permanent ponds (n = 81 larvae) within YNP, and fit von Bertalanffy models of growth [29] to each group (Figure 1). The calculated tangent of the model was higher for larvae from permanent ponds (L_∞ _= 75.1) than larvae from ephemeral ponds (L_∞ _= 58.7), reflecting the fact that the larvae from permanent reached larger sizes. The rate constants and x-intercepts were similar between both models.

**Figure 2:**
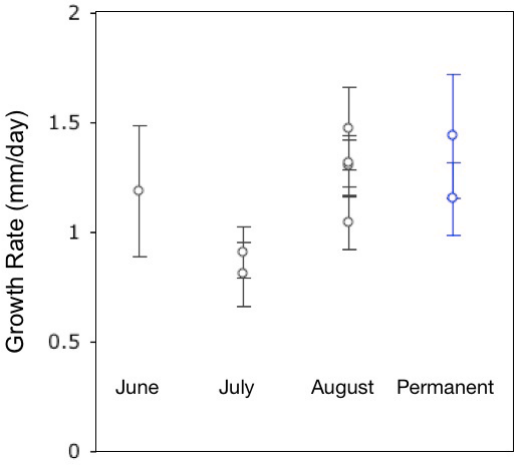
**Early growth rates are similar in ponds of different hydroperiods**. Slope of growth rates (mm/day) of YNP larval salamanders in 2008 prior to 30 June.

Modelled as linear functions, larval growth was statistically indistinguishable between the two environments (Permanent: y = 21.6 + 0.87x, r^2 ^= 0.72; Ephemeral: y = 18.2 + 0.87x, r^2 ^= 0.57). We used linear models to describe early growth rates during 2008 prior to 30 June before any of the ponds dried completely. Considering the nine ponds with sufficient sampling to create realistic linear models of early larval growth, we found slopes ranging from 0.81 (Pond 2, dry in July) to 1.48 mm/day (Pond 37, dry in August). Based on these nine locations, we found no statistical differences in early growth rate between Hydroperiod categories (Figure 2).

### Hatching dates and larval period

We used the nine independent models of early growth to infer the dates at which pond populations hatched in 2008. The projected hatching dates ranged from 1 June (Pond 2, dry in July) to 14 June (Pond 38, permanent; Table 1). The smallest metamorph identified during the course of this study was 45 mm SVL, and we interpret this size as close to the minimum size at which metamorphosis can occur (SVL_m_). Growing at the most rapid recorded linear growth rate (1.48 mm/day, Pond 37), it takes 33 days from hatching at 5 mm to reach SVL_m _(~45 mm).

**Table 1:** Characteristics of different ponds in 2006 through 2008

Pond	Elevation (m)	Maximum area (HA)	Latitude N	Longitude W	Growth rate in 2008	Projected date of hatching in 2008
**1**	1915	0.6	44°54'57.04"	110°23'49.10"	1.04	June 5

**2**	1914	1.4	44°54'51.42"	110°23'33.54"	0.81	June 1

**3**	1914	0.2	44°54'50.48"	110°23'27.38"	1.30	June 8

**4**	1914	1.4	44°54'43.53"	110°23'21.59"	1.19	June 8

**5**	1903	2.1	44°54'46.60"	110°23'03.77"		

**26**	1796	7.9	44°54'44.46"	110°21'00.79"	1.44	June 8

**33**	1919	1.2	44°55'07.96"	110°20'27.94"		

**34**	1896	1.1	44°55'11.25"	110°20'33.84"		

**36**	1901	2.8	44°55'52.73"	110°18'53.23"	1.32	June 9

**37**	1903	0.8	44°56'02.26"	110°18'49.12"	1.48	June 13

**38**	1856	1.2	44°55'49.08"	110°19'01.25"	1.15	June 14

**43**	1901	0.4	44°56'16.65"	110°18'40.05"	0.91	June 4

**47**	2177	9.3	44°57'37.64"	110°37'29.92"		

**49**	2134	0.1	44°55'55.41"	110°17'10.35"		

**Ice Lake**	1677	0.8	45°01'54.18"	110°45'01.78"		

**A**	1791	0.7	45°0'18.41"	110°42'14.59"		

**E**	1997	0.7	44°59'48.00"	110°43'26.21"		

**F**	1807	0.7	45°1'16.30"	110°44'5.59"		

**J**	2214	0.3	44°58'12.65"	110°37'19.66"		

**L**	2145	0.9	44°57'19.03"	110°30'10.00"		

**Rainbow**	1809	0.9	45°1'16.68"	110°44'17.60"		

**Axolotl 1**	2189	0.3	45°14.788'	111°52.871'		

**Axolotl 2**	2157	4.0	45°14.551'	111°53.002'		

**Axolotl 3**	2214	0.1	45°14.620'	111°52.801'		

2008 Growth Rate is the slope of the linear model of SVL prior to 30 June relative to date. 2008 Hatching Date is the date at which the linear model SVL = 5. All ponds are in YNP except Axolotl 1, 2, and 3.

Snowmelt and spawning occur in early April and hatching occurs approximately 55 days later in early June. Thus, in this region, a hydroperiod of more than 88 days (from April snowmelt to early July) is necessary to support this species through metamorphosis. Our model predicts that few if any individuals will reach metamorphosis before July. Indeed, the earliest young-of-the-year metamorph was captured on 11 July.

### Size of Larvae and Size at Metamorphosis

The average size of larvae from permanent ponds was more than 15 mm larger than the average size of larvae from ephemeral ponds. However, this comparison was biased by the fact that collection occurred earlier in ephemeral ponds. We employed a generalized linear mixed effects model [30] considering SVL of YNP larvae as a function of Year of Collection, Date of Collection and Hydroperiod, with random slopes for Date within Pond and Year within Pond (AIC = 6161, log likelihood = -3069). This model performed somewhat better than the same model without the Hydroperiod variable (AIC = 6170, log likelihood = -3076), but an ANOVA showed that the two models were not statistically different from one another. Thus, when Date of Collection and differences in growth between Ponds and Years were factored out, Hydroperiod had no measurable effect on larval size. Results were unchanged when Hydroperiod was assessed as a binary variable (permanent/ephemeral).

**Figure 3:**
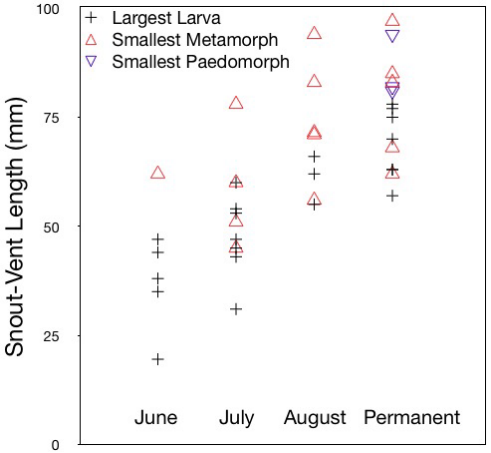
**Maximum larval size and minimum size at maturity increase with hydroperiod**. Each point represents size category for a single pond-year 2006-2008. Black crosses show SVL of largest larva found in each pond-year; red triangles show SVL of smallest metamorph found in each pond-year; inverted blue triangles show SVL of smallest paedomorph in each pond-year. Pond-years categorized by hydroperiod into locations that dried in June, July, August, and locations that did not dry (Permanent).

We compared the sizes of the largest larvae found in each pond during each year, including largest larvae from all permanent pond-years and largest larvae from ephemeral pond-years within three weeks of pond drying (Figure 3, black crosses). ANOVA revealed significant differences between Hydroperiod categories (df = 3, f = 3.0, p-value < 0.05), although Tukey honest significant differences showed no significance between the four categories. However, when the largest larvae from permanent ponds were compared to the largest larvae from ephemeral ponds (Hydroperiod categories June, July and August), larvae from permanent ponds were ~14 mm larger than larvae from ephemeral ponds (t = -2.6, df = 14.1, p-value = 0.02). Paedomorphs were found only in permanent ponds (Figure 3, purple inverted triangles).

### Allometry and shape change

**Figure 4:**
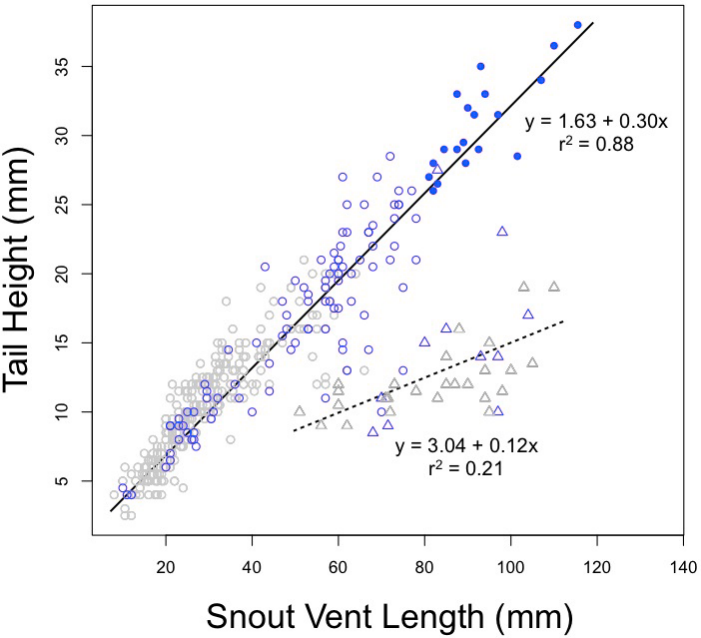
**Allometric relationships in aquatic and terrestrial salamanders**. Open circles represent larvae (n = 373); filled circles show paedomorphs (n = 19); open triangles show terrestrial individuals (n = 39). Blue symbols denote individuals collected from permanent locations; gray symbols denote those collected from ephemeral ponds. Solid line and adjacent formula indicate best fit linear model for all aquatic individuals (larvae and paedomorphs); dashed line and adjacent formula indicate the best fit line for all terrestrial individuals.

We performed a principal components analysis using the natural logarithms of eight different physical parameters (Table 2). All measurements loaded strongly onto PC1, with SVL contributing the strongest loading. PC2, the shape component, was dominated by tail height. Although PC2 explained relatively little of the total variance in shape (4.9%), PC2 along with PC1 strongly separated aquatic from terrestrial individuals (similar to Figure 4). Aquatic individuals (both larvae and paedomorphs) showed a negatively allometric relationship between SVL and tail height. Larvae and paedomorphs showed approximately a 3:1 relationship between SVL and tail height; this relationship changed to 8:1 after metamorphosis (Figure 4).

**Table 2:** Principal components analysis of salamander physiology

	PC1	PC2	PC3	PC4	PC5	PC6	PC7	PC8
**Snout vent length**	**-0.3671**	-0.1264	0.1101	-0.2088	0.0598	-0.3813	**0.7488**	-0.2897

**Average front leg length**	-0.3616	-0.1704	0.4020	-0.1325	0.3608	-0.0167	0.0585	**-0.7261**

**Average rear leg length**	-0.3622	-0.1919	0.3677	-0.0349	0.2899	0.3445	0.3381	0.5980

**Tail length**	-0.3553	-0.1429	0.2964	0.3521	**-0.7942**	0.0573	0.0186	-0.0841

**Tail height**	-0.3014	**0.9390**	0.1230	0.0522	0.0393	0.0789	-0.0434	0.0024

**Tail width**	-0.3533	-0.1059	-0.4626	**0.7332**	0.3285	-0.0410	-0.0454	-0.0286

**Head width**	-0.3652	0.0184	-0.2859	-0.2983	-0.1181	**-0.6130**	0.5482	0.0733

**Body width**	-0.3579	-0.0669	**-0.5407**	-0.4294	-0.1676	0.5725	-0.1298	-0.1339

**Standard Deviation**	2.6800	0.6253	0.3926	0.3232	0.3052	0.1790	0.1524	0.1431

**Proportion of Variance**	0.8980	0.0489	0.0193	0.0131	0.0116	0.0040	0.0029	0.0026

Loadings, standard deviations and proportion of variances of eight principal components. The largest loading factors for each component are shown in bold.

## Discussion

The *A. t. melanostictum *pond populations in this region of the GYE are closely related to one another, exchange gene flow, and exhibit low overall genetic diversity [31-33]. Therefore, majority of the phenotypic variation encountered in this region is due to phenotypic plasticity rather than genetic differentiation and adaptation. Measuring 609 individuals collected from 25 ponds within the GYE, we documented substantial variation in the duration of larval periods and the sizes at metamorphosis, differences which correlated with differing pond hydroperiods.

Larvae in more permanent environments reached greater sizes than larvae in ponds with brief hydroperiods (Figures 1 and 3). While most larvae from ephemeral ponds had either metamorphosed or perished before reaching 60 mm SVL, larvae in permanent environments were able to remain under aquatic growth conditions up to nearly 80 mm SVL, after which they either became paedomorphic or metamorphic. The smallest metamorph collected from a permanent environment was 62 mm SVL, 17 mm larger than the smallest metamorph found in an ephemeral environment (45 mm). Indeed nearly half of metamorphs collected from ephemeral environments were smaller than the smallest metamorph collected from any permanent environment. Furthermore, while the first emerging metamorph was captured from an ephemeral location on 10 July (Figure 1: b), the first metamorph emerged from a permanent pond more than a week later (and this individual was more than 80 mm SVL, suggesting that it may have not been young-of-the-year; Figure 1: c).

Many permanent environments contain over-wintering larvae, which postpone metamorphosis until their second or third years. Individuals under this developmental regime grow as aquatic larvae for several years, and some eventually forego metamorphosis to become reproductively mature neotenic paedomorphs ("paedomorph advantage") [21]. Only the two most stable environments examined, Ice Lake and Rainbow Lake, contained paedomorphs. These were the only two YNP locations that never dried over the course of the three year study period. We did not identify any small progenic paedomorphs (i.e. "best of a bad lot" paedomorphs [21]), possibly because the resource-poor locations that might have promoted precocious paedomorphosis were ephemeral and would have destroyed lingering aquatic individuals.

Larval growth rate determines the amount of time required to reach SVL_m_, and therefore defines the minimum larval period required prior to metamorphosis. Environmental conditions greatly influence larval growth rates in controlled settings [12,34]; however, we found average larval growth to be extremely similar between ponds and ponds of different hydroperiods. The earlier onset of metamorphosis observed in environments with shorter hydroperiods was not accompanied by accelerated larval growth rate. Between 16 June and 30 June, 2008 (during which all ponds were hydrated), individuals grew a linear average of more than 1 mm per day (Figure 2).

We used the early larval growth models to extrapolate dates of hatching in each of these ponds, all of which fell between 1 June and 14 June (Table 1). Assuming an incubation period of 55 days (the length of time between spawning and hatching in the permanent Ice Lake location [35]), we further extrapolated that spawning likely occurred in each of these ponds during the first two weeks of April, soon after snow melted from the region [36]. These inferred hatching dates are a month earlier than the hatching dates recorded at Ice Lake in 1993 [35]. Northern YNP has experienced warmer spring temperatures and earlier snowmelt dates in recent decades [28,36], suggesting that long-term warming has contributed to phenological changes and earlier breeding of this amphibian population [see also [37-39]].

**Figure 5:**
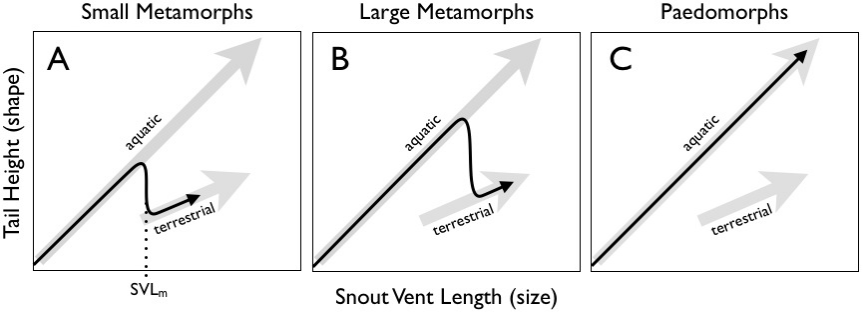
**Allometric relationships through ontogeny**. Aquatic and terrestrial individuals show different relationships between tail morphology (y axis) and size (x axis), represented by the two large grey arrows. A: Larvae in ephemeral environments undergo early metamorphosis and transform into terrestrial individuals at a small size. SVL_m _shows the minimum size for metamorphosis. B: Larvae in more permanent environments delay metamorphosis and transform into terrestrial adults at larger body sizes. C: In permanent aquatic environments, some larvae forgo metamorphosis to become reproductively mature neotenic paedomorphs.

At metamorphosis, amphibians undergo major restructuring of internal and external organ systems, remodelling somatic tissues to adopt a terrestrial lifestyle. In addition to the loss of external gills, the lateral tail swim fin is lost, resulting in a decrease in tail height. Tail height dominated the second component (shape) of the principal components analysis (PCA; Table 2), and tail height compared to size reliably separated aquatic from terrestrial individuals (Figure 4). After loss of the swim fin, terrestrial individuals showed far narrower tails relative to their length. Metamorphosis furthermore modified the relationship between size and tail shape. For every centimetre SVL growth gained by an aquatic salamander, it gained approximately 30 mm of tail height; while every centimetre of snout-vent growth gained by a terrestrial salamander was accompanied by only 12 mm of tail height. Paedomorphs maintained the aquatic SVL/tail height relationship throughout their lives. We have developed these observations into a conceptual framework of allometric relationships though different developmental pathways (Figure 5). As shown, the metamorphosis of an individual at a small size (Figure 5: A), at a large size (B) or not at all (C) is functionally determined by the hydroperiod of the pond environment.

Individuals that are able to delay metamorphosis (Figure 5: B) may be more likely to survive and reproduce than individuals that metamorphose at or close to SVL_m _(Figure 5: A) [5,18-20,40]. Although early metamorphosis or progenic paedomorphosis allow immediate reproduction [21,41], studies show that salamanders that delay metamorphosis to larger body sizes enjoy overall benefits in survival and fecundity [18,40]. Larger individuals are at less risk of desiccation and predation in terrestrial environments [12], are able to more efficiently capture terrestrial food, have higher mating success, larger clutch sizes and generally higher fecundity than smaller individuals [24,40]. As ponds in this region become increasingly ephemeral [28], resident amphibians may be forced to adopt ever more rapid and potentially less fecund developmental profiles.

Although differences in adult survival and fecundity have not yet been tested in this region, we determined that many ponds in the area are now entirely unsuitable for *Ambystoma *development, leaving the population vulnerable to continued warming. Larvae must reach a certain minimum size in order to undergo successful metamorphosis [19,21,24,42], and based on field observations, the SVL_m _for this population is close to 45 mm. If the hydroperiod is shorter than the minimum larval period required to reach SVL_m_, larvae have no opportunity to escape a drying pond. Growing at the most rapid documented growth rate, the minimum larval period is approximately 33 days from hatching, and the incubation period of eggs is approximately 55 days. Thus, these salamanders need a minimum of 88 days of pond hydration achieve SVL_m_. Snowmelt and egg deposition occur in early April, and ponds must therefore contain water through approximately the first week in July to support populations through metamorphosis. As predicted by this model, the first young-of-the-year metamorph was captured on 11 July. Earlier snowmelt [36] could extend the functional hydroperiod, potentially buffering the effects of earlier drying, however, such changes in seasonality may negatively impact the population in other ways [37-39].

Over the course of three years, we observed six locations to dry during the month of June, and hundreds of bodies of young salamanders were found trapped in the drying wetlands [28]. These ponds with hydroperiods shorter than the required larval period serve as reproductive sinks, where few if any larvae survive to recruitment. Larvae in ephemeral environments do not appear to accelerate larval growth rate, and this species may thus be poorly equipped to thrive in newly ephemeral, rapidly drying environments.

*A. t. melanostictum *is a long-lived, opportunistically breeding organism, and populations are able to recover from dry years [43]. Indeed, although past droughts likely contributed to population bottlenecks and loss of genetic diversity [31], the GYE population has recovered from both ancient climatic fluctuations [27] and more recent dry periods during the late 1980s and the early 2000s [28]. Nonetheless, long term drought conditions can prevent breeding over the course of decades, exceeding both the lifespan of the organism and the capacity of populations to recover.

## Conclusions

The adaptable, complex lifecycle of *A. t. melanostictum *allows populations to exploit a variety of hydrologically diverse habitats. Nonetheless, there are fitness costs associated with the rapid metamorphosis necessary for development in ephemeral environments. Even though the timing of metamorphosis is highly plastic in this species, we found that the rate of larval growth is consistent between environments, and that there is an absolute minimum amount of time required for larvae to reach the minimum size for metamorphosis. Several ponds in northern YNP now have hydroperiods too short to support the minimum larval period, and serve as reproductive sinks. If this landscape continues to dry and more locations become functionally uninhabitable, the population may be pushed beyond the limits of its adaptive and reproductive capacity.

## Methods

### Study area and collection

We focused this study on 22 bodies of water in northern YNP, as well as 3 lakes northwest of the northern park boundary but within the GYE (Axolotl 1, 2, and 3). These 25 locations represented a variety of developmental habitats for larval amphibians, ranging from small, ephemeral kettle ponds fed by local aquifers that dried early in the summer, to permanent ponds larger than seven hectares. Elevation and coordinates of each location was recorded using a handheld GPS unit; maximum area was estimated from satellite photos (characteristics and locations in Table 1). During every summer 2006-2008, each pond was characterized as having dried to 3 cm or less of standing water during June, July, August or never (permanent; see Additional file 1). Not all ponds were sampled every year of the study.

Each summer we captured and released between 1 and 52 (mean = 16.5) immature larval, paedomorph and terrestrial *A. t. melanostictum *individuals from in and around each sampling location. Most individuals were captured by active dip-netting, some were captured using aquatic funnel or terrestrial pitfall trapping. SVL of each individual was measured to the nearest mm. Individuals captured during 2007 and 2008 were also measured for limb length, tail length, tail height, tail width, head width and body width.

Metamorphic status was determined based on gill morphology: individuals with fully brachiated gills were considered larvae if they were small and immature and were considered paedomorphs if their external cloacae appeared mature. Individuals lacking gills or with gills in the process of resorption were considered to be terrestrial adults.

### Size and growth assessments

All analyses were performed in R 2.8.1 [44]. We assessed average growth of all larvae from the 22 ponds within YNP. Ponds outside of the park were excluded from growth rate analyses because they were at a higher elevation and spawned later in the year. Average growth was fit to the von Bertalanffy model of growth [11,29]:

where L(t) is size at time t, L_∞ _is the mean maximum size, t_0 _is the x-axis intercept and k is the rate constant. We used linear models to describe early growth (before 30 June) of nine individual ponds during 2008. We used these linear models to approximate the date at which the populations hatched in 2008. This species is generally 5 mm SVL at hatching, so we extrapolated the model to the date at which SVL = 5.

We fit the sizes of YNP larvae to a generalized linear mixed model using the lmer function in the R package lme4 [30]. This mixed model assessed larval SVL as it varied with the categorical variable Year, the categorical variable Hydroperiod (June, July, August, Never), and with Date of Collection as a continuous variable. Since observations of salamanders were clustered by Ponds and by Years within Ponds, we included a random intercept for Pond and a random interaction between Year and Pond. In order to capture the possibility of different slopes for date across ponds, we also included random slopes for Date within Ponds.

The largest larva from each pond-year was recorded for ephemeral ponds from which larvae had been collected within 3 weeks (21 days) of drying. Thus, the largest larva from Pond 34 in 2008 was excluded because the last larva was collected from that location more than 5 weeks before the pond dried. Largest larvae from permanent ponds were considered each year regardless of when individuals were collected. Smallest metamorphs and paedomorphs were determined for every pond-year.

To characterize differences in growth patterns through ontogeny, we plotted tail height against SVL. We performed a PCA using as variables the natural log-transformed measurements of SVL, average front leg length, average rear leg length, tail length, tail height, tail width, head width and body width.

## Abbreviations

(GYE): Greater Yellowstone Ecosystem; (YNP): Yellowstone National Park; (SVL): Snout vent length; (PCA): Principal components analysis.

## Authors' contributions

EAH and SKM designed the study. SKM collected specimens, analyzed data and prepared the manuscript. Both authors have read and approved the final manuscript.

## Supplementary Material

Additional file 1 **Hydroperiods and collection dates**. Total number of *A. t. melanostictum *individuals (larvae, metamorphs and paedomorphs) collected each day shown for every pond-year. Approximate hydroperiods (± 7 days) are shown in light blue; permanent ponds are blue throughout. Individuals collected outside of the hydroperiod represent metamorphs collected from the location after drying. Click here for file
