# Supplementary material for: Developmental dynamics of Ambystoma tigrinum in a changing landscape
Source: BMC Ecol. 2010 Apr 3;10:10. doi: 10.1186/1472-6785-10-10 (PMC2861013; doi:10.1186/1472-6785-10-10)
Supplement: Additional file 1 — Hydroperiods and collection dates. Total number of A. t. melanostictum individuals (larvae, metamorphs and paedomorphs) collected each day shown for every pond-year. Approximate hydroperiods (± 7 days) are shown in light blue; permanent ponds are blue throughout. Individuals collected outside of the hydroperiod represent metamorphs collected from the location after drying. [file 1472-6785-10-10-S1.PDF]

Pond	Year	Total	June															July																					
			16	17	18	19	20	21	22	23	24	25	26	27	28	29	30	1	2	3	4	5	6	7	8	9	10	11	12	13	14	15	16	17	18	19	20	21	22
**1**	2006	37																											18				19						
**1**	2007	**2**						2																															
**1**	2008	28	13					4				5				6																							
**2**	2008	22	9					3				5				5																							
**3**	2008	22						10				6				6																							
**4**	2008	16						4				7				5																							
**5**	2006	21																									21												
**5**	2008	17							6			6			4				1																				
**26**	2007	25														9															8							4	
**26**	2008	14						10					4																										
**33**	2007	5															4															1							
**33**	2008	40	5													3	11	21																					
**34**	2006	10																					10																
**34**	2007	**6**															6																						
**34**	2008	12	2														9	1																					
**36**	2007	18							1				1		7	1														2									
**36**	2008	21		10									5				6																						
**37**	2007	2													1				1																				
**37**	2008	20		6					5				4				5																						
**38**	2006	8														3	1																	3			1		
**38**	2007	9								1							1		1																				
**38**	2008	11			3				1		1					5			1																				
**43**	2008	16		11											5																								
**47**	2007	15																					1																
**45**	2007	1																1																					
**49**	2006	**52**													4									11						37									
**Ice Lake**	2007	32																																	1			1	
**Ice Lake**	2008	16			2	1	1	1	1			3	1	6																									
**A**	2007	11																11																					
**E**	2007	1																		1																			
**F**	2007	21																						3			4	5											
**J**	2007	19												4																			9						6
**J**	2008	10								5								5																					
**L**	2008	4					4																																
**Rainbow**	2007	12																																					
**Axolotl 1**	2007	11																																					
**Axolotl 2**	2007	12																																					
**Axolotl 3**	2007	11																																					

Pond	Year										August																									
		23	24	25	26	27	28	29	30	31	1	2	3	4	5	6	7	8	9	10	11	12	13	14	15	16	17	18	19	20	21	22	23	24	25	Permanent
**1**	2006																																			
**1**	2007																																			
**1**	2008																																			
**2**	2008																																			
**3**	2008																																			
**4**	2008																																			
**5**	2006																																			
**5**	2008																																			
**26**	2007									1	1	1																		1						
**26**	2008																																			
**33**	2007																																			
**33**	2008																																			
**34**	2006																																			
**34**	2007																																			
**34**	2008																																			
**36**	2007					2																									3	1				
**36**	2008																																			
**37**	2007																																			
**37**	2008																																			
**38**	2006																																			
**38**	2007					1	1							1													2				1					
**38**	2008																																			
**43**	2008																																			
**47**	2007																											2	10	2						
**45**	2007																																			
**49**	2006																																			
**Ice Lake**	2007	2	1			1	1	4	9	5	7																									
**Ice Lake**	2008																																			
**A**	2007																																			
**E**	2007																																			
**F**	2007			5																4																
**J**	2007																																			
**J**	2008																																			
**L**	2008																																			
**Rainbow**	2007			1												1	1	1	3	2	3															
**Axolotl 1**	2007																																11			
**Axolotl 2**	2007																																	10	2	
**Axolotl 3**	2007																																	11		
